# Nanoparticles as Vaccines to Prevent Arbovirus Infection: A Long Road Ahead

**DOI:** 10.3390/pathogens10010036

**Published:** 2021-01-05

**Authors:** Gabriel Augusto Pires de Souza, Raíssa Prado Rocha, Ricardo Lemes Gonçalves, Cyntia Silva Ferreira, Breno de Mello Silva, Renato Fróes Goulart de Castro, João Francisco Vitório Rodrigues, João Carlos Vilela Vieira Júnior, Luiz Cosme Cotta Malaquias, Jônatas Santos Abrahão, Luiz Felipe Leomil Coelho

**Affiliations:** 1 Laboratório de Vacinas, Departamento de Microbiologia e Imunologia, Instituto de Ciências Biomédicas, Universidade Federal de Alfenas, Alfenas 37130-001, Brazil; neogaps@microb.dout.ufmg.br (G.A.P.d.S.); renato_froes_goulart@hotmail.com (R.F.G.d.C.); joaofvrodrigues@gmail.com (J.F.V.R.); carlosvvieira@hotmail.com (J.C.V.V.J.); luiz.malaquias@unifal-mg.edu.br (L.C.C.M.); 2 Laboratório de Vírus, Departamento de Microbiologia, Instituto de Ciências Biológicas, Universidade Federal de Minas Gerais, Belo Horizonte 31270-901, Brazil; jonatas.abrahao@gmail.com; 3 Laboratório de Virologia Básica e Aplicada, Departamento de Microbiologia, Instituto de Ciências Biológicas, Universidade Federal de Minas Gerais, Belo Horizonte 31270-901, Brazil; raissa.biotec@gmail.com; 4 Núcleo de Pesquisas em Ciências Biológicas, NUPEB, Universidade Federal de Ouro Preto, Ouro Preto 35400-000, Brazil; ricardolemesg@gmail.com (R.L.G.); csf.ferreira@gmail.com (C.S.F.); breno@ufop.edu.br (B.d.M.S.)

**Keywords:** arbovirus, experimental roadmap, nanoparticles, vaccine

## Abstract

Arthropod-borne viruses (arboviruses) are a significant public health problem worldwide. Vaccination is considered one of the most effective ways to control arbovirus diseases in the human population. Nanoparticles have been widely explored as new vaccine platforms. Although nanoparticles’ potential to act as new vaccines against infectious diseases has been identified, nanotechnology’s impact on developing new vaccines to prevent arboviruses is unclear. Thus, we used a comprehensive bibliographic survey to integrate data concerning the use of diverse nanoparticles as vaccines against medically important arboviruses. Our analysis showed that considerable research had been conducted to develop and evaluate nanovaccines against Chikungunya virus, Dengue virus, Zika virus, Japanese encephalitis virus, and West Nile virus. The main findings indicate that nanoparticles have great potential for use as a new vaccine system against arboviruses. Most of the studies showed an increase in neutralizing antibody production after mouse immunization. Nevertheless, even with significant advances in this field, further efforts are necessary to address the nanoparticles’ potential to act as a vaccine against these arboviruses. To promote advances in the field, we proposed a roadmap to help researchers better characterize and evaluate nanovaccines against medically important arboviruses.

## 1. Introduction

### 1.1. Nanoparticles

**Figure 1:**
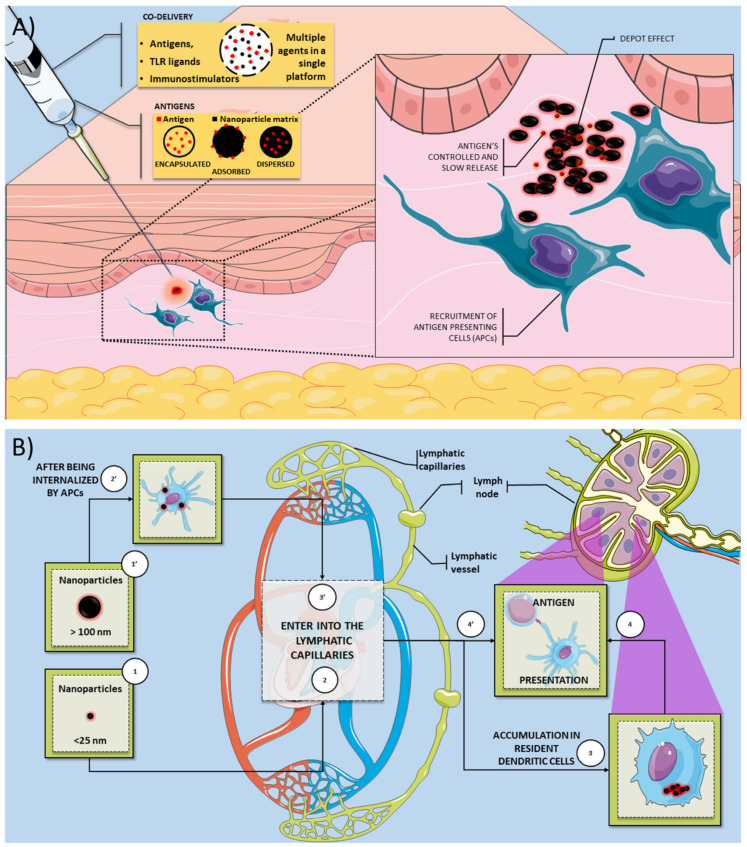
Overview of the mechanisms by which nanovaccines can induce an immune response. (**A**) Nanoparticles can be used as a vaccine platform for several infectious diseases since they can deliver antigens and several immunostimulatory molecules (TLR ligands and adjuvants). The antigen could be encapsulated, adsorbed, and dispersed on the nanoparticle matrix. The immunostimulatory activity of nanovaccines is related to several mechanisms, such as the depot effect, gradual release of vaccine antigens, and recruitment of antigen-presenting cells. (**B**) Smaller nanoparticles (<25 nm) are transported through the lymphatic system more quickly than larger particles (>100 nm). Smaller nanoparticles could accumulate in dendritic cells (DC) resident in the lymph nodes. These resident DC can process and present the antigen to T cells on the lymph node. In contrast, larger nanoparticles are efficiently uptake by APCs present or recruited on the injection site. These APCs can also process the antigen and migrate to the lymph node to present the T cells’ antigen.Legend: APC: antigen-presenting cell; DC: dendritic cells; TLR: toll-like receptor.

Nanoparticles (NPs) are defined as particulate dispersions or solid particles with a size between 10–1000 nm that demonstrate unique properties and functions due to their size [1,2,3]. Although nanotechnology is not a new concept, it has gained significant prominence in recent decades due to advances in materials science and nanoengineering, making it especially attractive for bioscience applications, such as drug and antigen delivery systems [4,5,6]. Nanoparticles based on organic and inorganic compounds have been widely explored as new vaccine platforms due to their ability to stimulate the immune system and provide sustained antigen release after vaccine administration [4,7,8]. Several studies have described the adjuvant properties of nanoparticles, once they can co-deliver multiple agents in a single biocompatible platform, which can improve absorption and efficiency compared to conventional treatment [9,10]. Nanoparticles can also provide a controlled and slow release of antigens, creating a depot at the administration site providing potential protection against antigen degradation [11,12] (Figure 1A).

Nanoparticles have been extensively explored as new vaccines because they allow the antigens to be encapsulated, adsorbed, or dispersed in the nanoparticle’s matrix [1,13]. The nanoparticle matrix components’ choice is essential to modulate the antigen’s release during transport and at the injection site [2]. The biopolymers used on biodegradable nanoparticles can be natural, such as proteins and carbohydrates, or synthetic origin [14,15]. In general, natural biomaterials could have some advantages, such as biocompatibility, biodegradability, and low immunogenicity [16]. In addition to the various material possibilities for constructing nanoparticles, physical–chemical parameters (size, charge, and morphology) must be evaluated to optimize the nanoparticles’ functionality. Nanoparticle size can influence the in vivo distribution, toxicity, and the nanoparticles’ ability to act on the target cell and tissue [2]. For example, it has been reported that smaller nanoparticles (<25 nm) are transported through the lymphatic system more quickly than larger particles (>100 nm). Therefore, smaller nanoparticles could accumulate in dendritic cells in the lymph nodes (Figure 1B) [13]. Free versus cell-associated drainage of nanoparticles has a crucial effect on targeting cell populations by the differently sized particles [17,18]. Vaccine delivery systems with similar virus dimensions will facilitate the direct interaction of antigens with follicular B cells [17,19]. The natural drainage of nanoparticles associated with antigens to lymph nodes may not be a prerequisite for the induction of B cell responses. However, it facilitates and enhances B cells’ interaction with their cognate antigen and associated TLR ligands [19]. It was suggested that nanoparticle with 20–200 nm range are most effective to induce immune cell activation [19]. The shape and size of the nanoparticles also have a strong impact on activation of immune response. This knowledge should be considered for rationally design and develop next-generation vaccines against pathogens. For example, an in vivo study report that spherical ovalbumin particles (193 nm in diameter) produced a Th1-biased response, whereas rod-shaped ovalbumin particles (1530 nm in length) produced a Th2-biased response [20]. 

Besides the shape and size, other properties, such as the surface charge, can be manipulated to achieve the desired benefits [13]. The surface charge of a nanoparticle is generally characterized by its zeta potential, reflecting the electrical potential. This parameter is influenced by the composition of the particle and the medium in which it is dispersed. The zeta potential can also be used to determine whether a loaded active material is encapsulated in the nanoparticle matrix or adsorbed on their surface [2]. While positively charged NPs can interact using non-specific interactions with the negatively charged phospholipid components of the cell membrane [21], negatively charged nanoparticles may represent a better strategy for targeting nanoparticles because they eliminate the non-specificity of the charge-load interaction [22,23]. Negatively charged NPs can also bind to cells using specific interactions, such as cellular receptors.

The successful translation of nanoparticles to the clinic requires developing a simple, safe, cost-effective, and eco-friendly mode of synthesis. A better understanding of the safety mechanisms, biodistribution, and pharmacokinetics of NPs are also required. Additionally, it is necessary to understand the costs associated with scale production and verify if the technology is economically viable for the industry [24]. Although nanoparticles’ potential to act as new vaccines against infectious diseases has been identified [1,25,26], nanotechnology’s impact on developing new vaccines to prevent medically important arboviruses is unclear. In this context, it remains poorly understood if nanoparticles carrying arbovirus antigens can induce a protective immune response to these antigens. Thus, we used a comprehensive bibliographic survey to integrate data concerning the use of diverse nanoparticles as vaccines against medically important arboviruses. The methodology used for the search in the literature, extraction, and management of data and networks’ construction is presented in the Supplementary Materials.

### 1.2. Arboviruses

Arthropod-borne viruses (arboviruses) are a major public health problem worldwide, especially in tropical and subtropical countries. The emergence of several arboviruses in diverse geographic regions has attracted the World Health Organization (WHO) attention and research around the world. Several outbreaks of medically important arboviruses have been described in the last decade, with millions of people affected in different countries [27,28]. Several arbovirus species and the viruses belonging to the *Flaviviridae* and *Togaviridae* families are the most frequent arboviruses that infect humans. Chikungunya virus (CHIKV), Dengue virus (DENV), and Zika virus (ZIKV) are transmitted to humans in urban cycles through *A. aegypti* mosquitoes and rarely cause mortality. However, the high number of annual cases and debilitation of some infected people make these diseases an economic and worldwide health problem [29]. Yellow fever virus (YFV) is also transmitted by *Aedes aegypti* (urban cycle), and *Aedes africanus*, *Haemagogus,* and *Sabethes* mosquitoes (sylvatic cycle), and this arbovirus have a high mortality rate. There is also an epizootic transmission of some arboviruses from animal reservoirs to humans. Japanese encephalitis virus (JEV), Venezuelan equine encephalitis virus, West Nile virus (WNV), Rift Valley fever virus (RVFV), Oropouche virus (ORPV), and Mayaro virus (MAYV) are examples of arboviruses that can usually infect humans and cause severe symptoms such as encephalitis [30,31].

Vector control is the most used measure against arboviruses. However, this strategy fails in several countries due to vector diversity, uncontrolled urbanization, and increasing resistance to insecticides [32]. Therefore, vaccination is considered one of the most effective ways to control arbovirus diseases in the human population [33]. While vaccines could potentially prevent arbovirus infection in humans, there are few licensed vaccines. The most successful case for arbovirus mass immunization is the use of vaccines to prevent yellow fever. This vaccine is a live-attenuated vaccine that induces a high percent of seroconversion (95%) on recipients after a single dose. However, some severe reaction cases after vaccination, such as viscerotropic infection, were described [34,35]. In contrast, there are other human diseases caused by arboviruses that do not have vaccines. Dengue is usually considered one of the biggest concerns about arboviruses, as it is estimated that around 390 million people are affected by dengue every year [36]. However, other arboviruses have been gaining attention. For example, we could cite ZIKV once infection with this arbovirus could be associated with neurological disorders in adults and newborns [37,38].

This leading role in infections caused by the DENV and ZIKV is also seen in vaccines’ development. In addition to the YFV that had its vaccine developed in the last century [39], the DENV is the only one that has a licensed vaccine and two more vaccine candidates in an advanced stage of development. It is estimated that 40 to 60 institutions worldwide work on around 20 ZIKV vaccine candidates adopting different strategies, such as inactivated viruses, VLPs, recombinant viruses, and DNA vaccines. Some of these have already reached phase II trials, although this virus only came into evidence in 2015 [33].

For dengue disease, the CYD-TDV vaccine (Dengvaxia®—Sanofi Pasteur, Lyon, France) was approved for use in several countries. The World Health Organization (WHO) established that CYD-TDV is immunogenic and safe in seropositive individuals due to clinical trial data. However, this vaccine’s use on seronegative individuals could increase the risk of induction of severe dengue in those individuals due to an increased risk of antibody-dependent enhancement [40,41]. To date, several approaches have been developed toward generating vaccines for the other arboviruses, including live-attenuated strains, inactivated strains, subunit, RNA DNA, and recombinant proteins. However, most of these vaccine candidates are still in preclinical or clinical trials. Many of the vaccines that are under development or phase I trials were based on purified subunits, recombinant proteins, or other microbial components that are generally safe [29,41,42]. However, these antigens could be poorly immunogenic and therefore need the use of adjuvants and/or delivery systems to induce optimal immune responses [7,43].

## 2. Nanovaccines against Arboviruses

**Figure 2:**
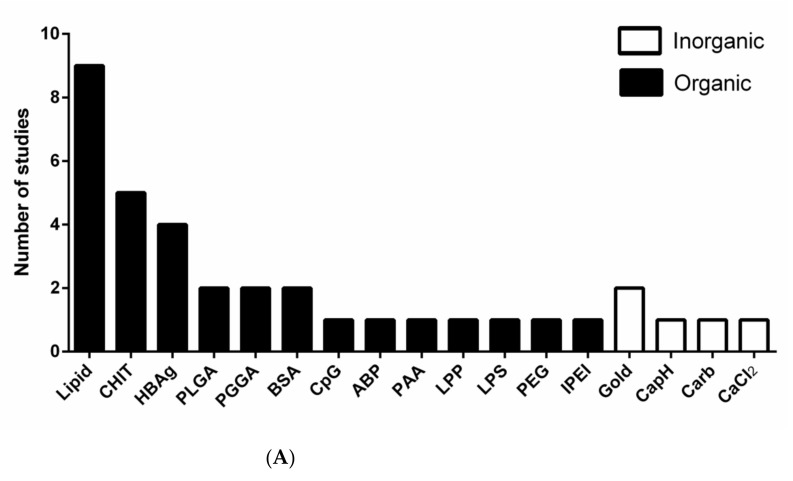

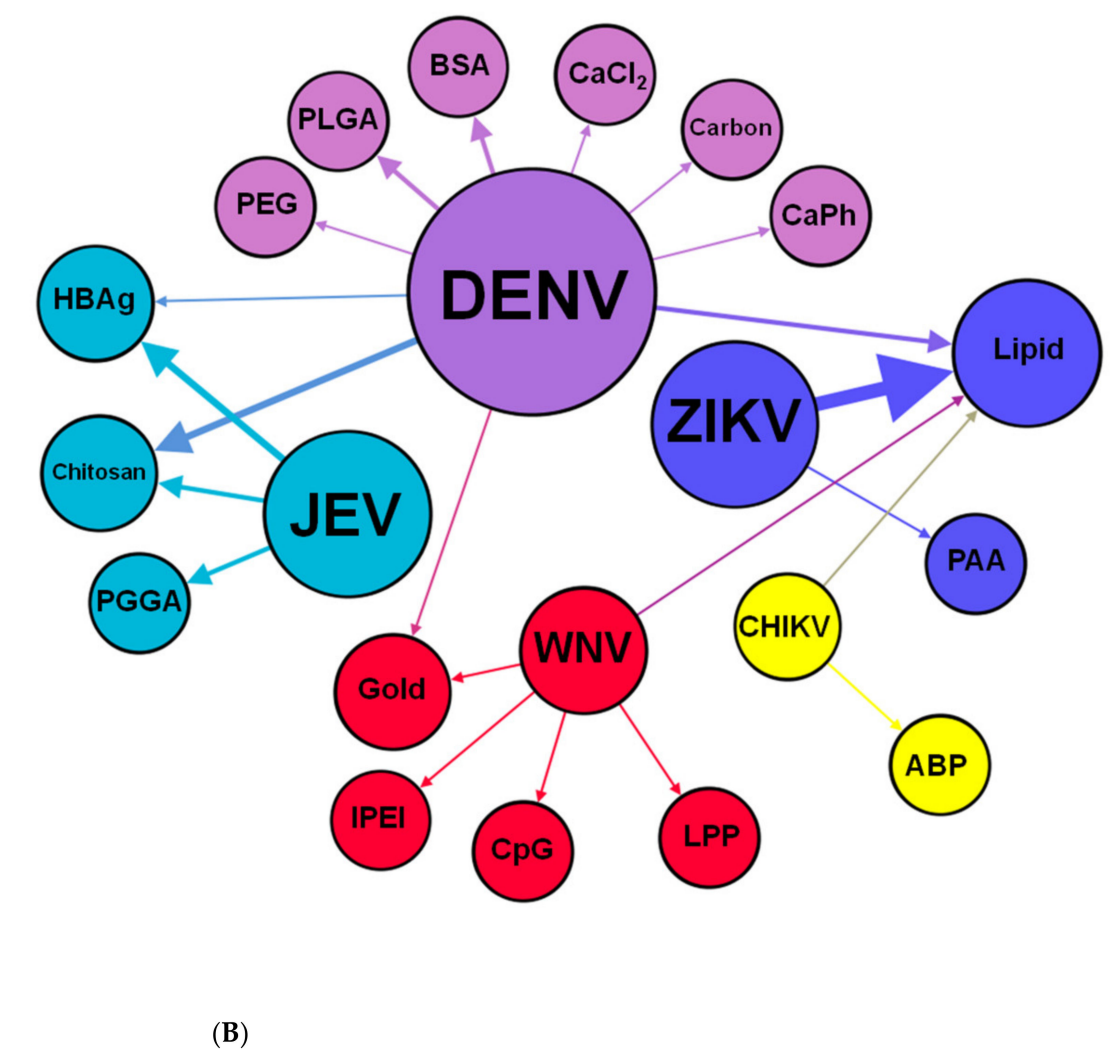
Nanoparticles as vaccines against medically important arboviruses. (**A**) Types of materials used to develop vaccines based on nanotechnology against DENV, ZIKV, JEV, WNV, and CHIKV. (**B**) Bipartite network graph showing a spatially connected network among the type of material used to develop nanoparticles and the target virus. Each node represents a virus or the type of nanoparticle material. The layout was generated using a force-based algorithm followed by manual rearrangement for better visualization of the connections. Legend: ABP: Amyloid beta-protein; BSA: bovine serum albumin; CaCl2: Calcium chloride; CapH: Calcium phosphate; Carb: carbon; CHIKV: Chikungunya virus; CHIT: chitosan; CpG: CpG oligodeoxynucleotide; DENV: Dengue virus; HBAg: Hepatitis B antigen; IPEI: polyethyleneimine; JEV: Japanese encephalitis virus; LPP: lipoprotein; LPP: lipoprotein; LPS: lipopolysaccharide; PAA: poly(amido amine), PEG: Polyethylene glycol; PGGA: poly(gamma-glutamic acid); PLGA: poly(lactic-co-glycolic acid); WNV: West Nile virus; ZIKV: Zika virus.

The materials used to develop nanoparticles as antigen delivery systems/vaccines against arboviruses are diverse. In general, organic polymers were preferentially used to develop nanovaccines against arboviruses compared to inorganic polymers (Figure 2A). Lipid nanoparticles (LNPs) are one of the most used in experimental vaccines against arboviruses. LNPs have been used as a delivery system. This could be attributed to some advantages of these nanoparticles, such as high mono dispersion, long time stability, and relatively good thermal stability [44]. LNPs generally consist of four components, (1) an ionizable cationic lipid, which promotes self-assembly in particles about 100 nm in size and allows the release of the antigen; (2) a lipid-bound polyethylene glycol (PEG), which increases the half-life of formulations; (3) cholesterol, a stabilizing agent; and (4) naturally occurring phospholipids, which support the lipid bilayer structure [45]. LNPs also promote improved protein stability, prevent proteolytic degradation, and have low toxicity since LNPs production do not need to use potentially toxic organic solvents, which can also have a harmful effect on antigens [46]. Currently, LNPs are one of the most used vectors for RNA delivery in vivo, especially for the treatment of genetic conditions, but some works explore them as vaccines [45,47,48].

Among inorganic nanoparticles, it is not surprising that gold nanoparticles (Au-NPs) are the most used in the production of vaccines against arboviruses (Figure 2A). As they were widely disseminated throughout nanotechnology, they are used in almost all medical applications (diagnostics, therapy, prevention). They are usually the most used material in inorganic nanoparticles for vaccine purposes [25,49]. Au-NPs could increase antigen stability by protecting them from premature degradation by proteolytic enzymes [50]. These NPs can induce a robust host immune response when used for the delivery of viral antigens such as influenza, in which the immobilization of the antigens on Au-NPs showed to be vital for inducing high levels of antibody response and also in provided complete protection against lethal influenza virus challenge in mice [51]. Surface-engineered Au-NPs were used in a DNA vaccine candidate against human immunodeficiency viruses. The results showed that this nanovaccine could significantly promote cellular and humoral immunity and T cell proliferation in vivo [52]. 

To better represent the use of nanoparticles as vaccines to prevent arbovirus infection it was built a bipartite network graph composed of 21 nodes all connected by 22 edges where the thickness of the edges represents the weight of interaction (Figure 2B). Therefore, edges with high thickness mean that this material was used more times to develop nanoparticles as vaccines against these arboviruses. Several types of materials were used to develop vaccine-based nanoparticles. For dengue vaccines, it seemed that several types of organic (bovine serum albumin, lipid) and inorganic materials (calcium chloride, carbon, and calcium phosphate) were used [33,53,54,55,56,57,58,59,60,61]. For ZIKV vaccines, only lipid and poly(amidoamine) nanoparticles were used [62,63,64,65,66,67], and for JEV vaccines, protein-VLPs, chitosan, and poly(gamma-glutamic acid) nanoparticles were tested [68,69,70,71,72,73,74,75]. Regarding the WNV vaccines, the materials tested were gold, CpG oligodeoxynucleotide, polyethyleneimine, lipoprotein, and lipid-based nanoparticles [76,77,78,79,80]. Lipid and amyloid based nanoparticles were tested for CHIKV vaccines [81,82]. It was noted that lipid nanoparticles were the most used technology since many studies aimed to develop nanoparticles using this material as new vaccines against DENV [56,61], ZIKV [62,63,64,65,66], and CHIKV [81]. Chitosan nanoparticles were also a systematic approach to develop nanovaccines against DENV [83,84,85] and JEV [73,75]. The VLPs made by Hepatitis B virus proteins were also tested on DENV [86] and JEV [73,74,75].

### 2.1. Type of Antigens Used on Nanovaccines against Arboviruses

DNA, RNA, VLPs, inactivated viruses, recombinant viral vectors, peptides, and subunit vaccines are used as experimental vaccines against arboviruses. Besides these several approaches, subunits-based vaccines are the most used [33,54,55,58,59,60,76,77,78,79,83,87]. Subunit vaccines are developed from selected fragments of the pathogen, such as proteins or polysaccharides. As advantages, they have fewer adverse reactions than live or inactivated whole vaccines but can often be poorly immunogenic. Therefore, subunit vaccines are often associated with adjuvants to lead to a more effective response [7,88]. Therefore, associating subunit vaccines against arbovirus with nanoparticles can represent an interesting strategy to obtain ideal immune responses and consolidate nanoparticles as adjuvants. The main advantages of nanovaccines are related to their intrinsic adjuvant activity and also to their ability to be easily uptake by antigen-presenting cells [9,10]. Additionally, their capacity to protect antigens and other molecules from degradation is an advantage [11,12]. The successful case of the Covid-19 vaccine using lipid nanoparticles to deliver the SARS-COV-2 spike mRNA strengthens the potential of nanoparticles to be used as promising platforms for infectious diseases vaccines [89,90,91].

In addition to subunit vaccines, RNA vaccines were also widely used as a promising strategy to develop nanovaccines against arbovirus. The use of mRNA vaccines has many advantages over subunit vaccines, dead and live-attenuated viruses, and DNA-based vaccines. The first of these is safety since mRNA is not an infectious or integrating platform. For this reason, it does not represent a potential risk of infection or insertion mutagenesis. Additionally, mRNA is degraded by normal cellular processes, and the use of modification and delivery methods can regulate its half-life. Finally, this type of vaccine production is fast and scalable to manufacture since high-performance in vitro transcription reactions can be performed [45]. Similar tosubunit vaccines, RNA vaccines are often associated with delivery systems. 

**Figure 3:**
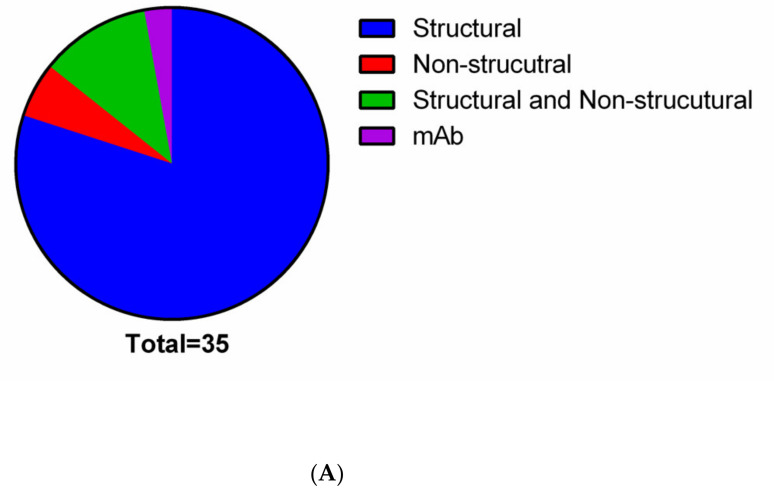

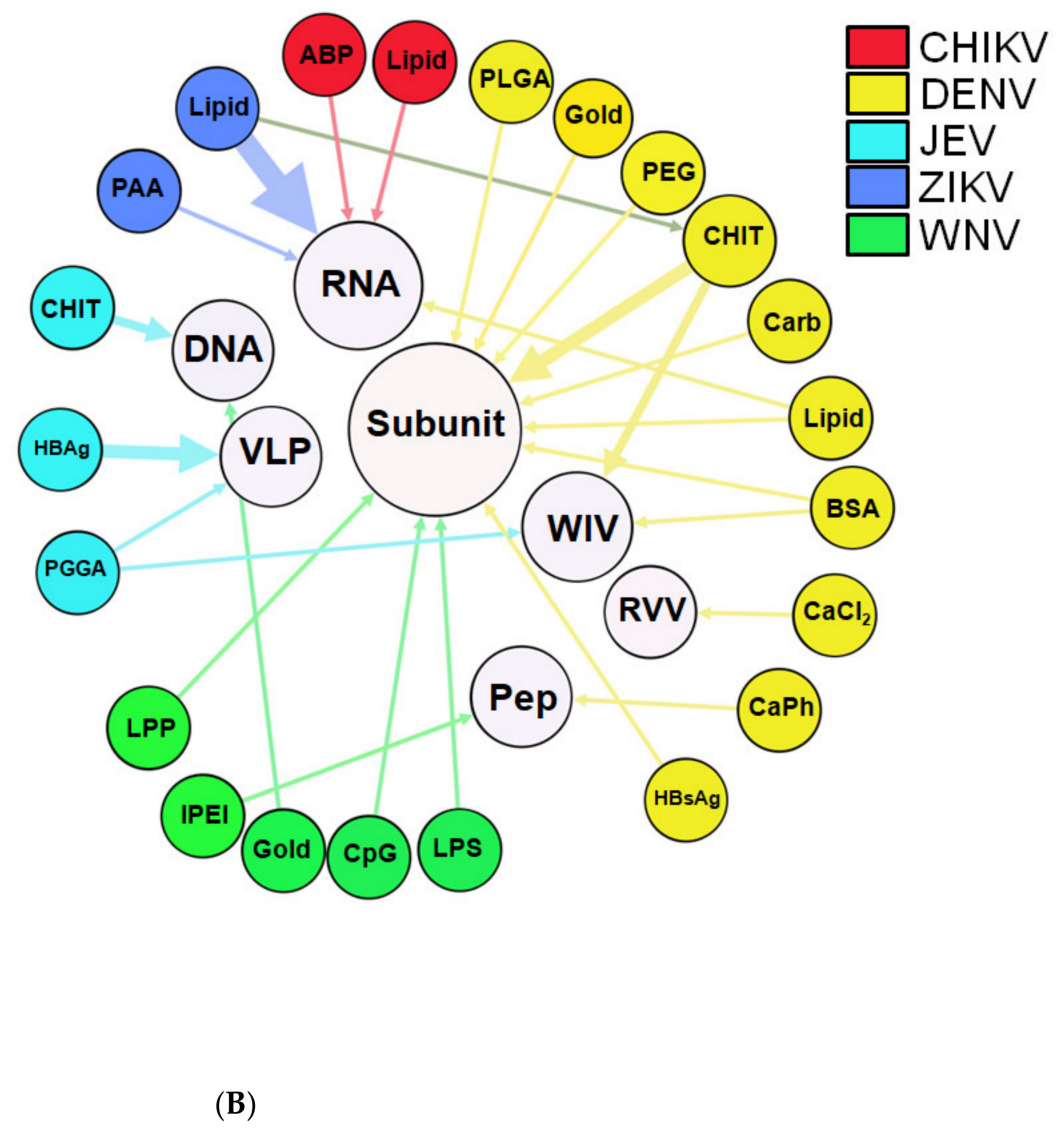
Virus antigens and nanoparticle network. (**A**) Use of structural and non-structural proteins of medically important arboviruses to develop vaccine-based nanoparticles. (**B**) Bipartite network graph showing a spatially connected network among the type of material used to develop nanoparticles and the vaccine approaches. Each node represents a type of nanoparticle material or the vaccine approach used. The nodes’ diameter is proportional to the edge degree. The layout was generated using a force-based algorithm followed by manual rearrangement for better visualization of the connections. Legend: ABP: Amyloid beta-protein; BSA: bovine serum albumin; CaCl2: Calcium chloride; CapH: Calcium phosphate; Carb: carbon; CHIKV: Chikungunya virus; CHIT: chitosan; CpG: CpG oligodeoxynucleotide; DENV: Dengue virus; HBAg: Hepatitis B antigen; IPEI: polyethyleneimine; IV: Whole inactivated virus; JEV: Japanese encephalitis virus; LPP: lipoprotein; LPP: lipoprotein; LPS: lipopolysaccharide; PAA: poly(amido amine); PEG: Polyethylene glycol; Pep: Peptide; PGGA: poly(gamma-glutamic acid); PLGA: poly(lactic-co-glycolic acid); RVV: recombinant viral vector; VLP: Virus-like particles; WNV: West Nile virus; ZIKV: Zika virus.

Among the type of viral antigen, most tested nanoparticles deliver structural proteins while few nanovaccines (5.7%) deliver non-structural proteins and or both types of antigens (Figure 3A). One study uses a lipid-encapsulated mRNA encoding a neutralizing human monoclonal antibody against CHIKV. Structural proteins of viruses are the preferred targets in most of the proposed nanovaccines against arboviruses. This could be due to their potential to induce neutralizing and long-lasting antibodies and memory cells [92,93,94]. Non-structural proteins from viruses can also represent interesting immunization strategies against arboviruses. Vaccines in their composition of the NS1 protein can be protective against several different flavivirus species [95,96,97]. The first report of a ZIKV vaccine, based on NS1 protein applied as a single intramuscular dose using an intracerebral lethal challenge model in immunocompetent mice, appeared to confer robust cellular and humoral responses. It provided 100% protection against ZIKV infection [98]. Another study suggests that incorporating NS1 and prM/M proteins on vaccine formulation are important to provide effective protection to the ZIKV [95].

As shown in Figure 3B, most of the studies used nanoparticles to deliver subunit antigens. However, only studies evaluated the nanoparticles’ vaccine potential to induce an effective immune response to DENV [33,54,55,56,59,83] and WNV [76,77] used subunit antigens. In general, the subunit antigens used on these nanoparticles were structural proteins (membrane and envelope proteins) produced and purified in a heterologous expression system (*E. coli,* mammalian, and insect cells) [54,55,58,59,60,76,77,78,79,83,87]. As shown in Figure 3B, nanoparticles carrying RNA were tested on ZIKV [62,63,64,66,67], CHIKV [81], and DENV [61] in preclinical immunization assays. Other types of antigens (DNA, peptide, inactivated virus, and recombinant viral vector) were also associated with nanoparticles, however, to a lesser degree than subunits or RNA antigens [53,57,69,72,73,80,82,84,85]. 

### 2.2. The Immune Response Induced by Nanovaccines against Arbovirus

The main objective of all studies that aimed to develop nanovaccines against arboviruses is to evaluate the activation of an immune response after immunization in vivo. As expected, most of the studies used mice as the animal model to nanoparticles’ effect on antibody production and T cell activation after immunization [56,58,61,77,85]. Few studies used alternative animal models such as non-human primates and guinea pigs. Mice are naturally resistant to infection by several flaviviruses, and this intrinsic characteristic impairs the evaluation of vaccine candidates on this model. An alternative to this inconvenience is the use of knockout animals in IFN receptor (IFNR) type I, since these mice became susceptible to flaviviruses infection [99,100,101]. However, the lack of IFN signaling impairs the response to a vaccine and makes it difficult to study the immune response induced after vaccine administration [46,100,101]. More recently, it has been established that mice with conditional knockout of IFNR type I expression in different immune system cells demonstrated a better ability to obtain an immune response than conventional immunocompromised mice [102,103]. Therefore, the use of this model could provide information about immune-protection and also be useful for tracking vaccine and nanovaccines candidates 

Moving from the mouse model to non-human primates (NHPs) models is essential as the next step before clinical testing. NHPs are especially interesting in arboviruses because they are also natural hosts and reservoirs for these viruses in endemic areas [104]. However, in some cases, the flavivirus infection does not induce any clinical disease even in the presence of a detectable viremia [105,106,107,108]. The quantification of viremia and antibodies and the possibility to evaluate antibody-dependent enhancement (ADE) on this model can generate useful data for the flaviviruses vaccine development [104,105]. On the other hand, working with NHPs requires strict regulation and the highest priority regarding animal welfare, which makes working with these animals arduous and expensive [106]. 

In general, animal models immunized with nanoparticles showed an increase of antibody levels after the immunization. Although it is desirable to achieve seroconversion after a single nanoparticle dose administration, most experimental studies were carried out with immunization regimens with one or two booster doses. Only 25% (9/35) of the studies used a single dose regimen and, in their majority, (6/9) using lipid nanoparticles as the basis for their vaccine platforms (Supplementary Table S1). Most studies measured IgG, but some measured IgM and IgA against viruses’ antigens. A few studies do not provide any information about the antibody class measured [53,54,76,82,83]. Almost all studies showed neutralizing antibodies against infectious viruses and some showed non-neutralizing activity or did not perform plaque reduction neutralization assays. Concerning cellular immune response, a Th1 response was induced by almost all studies. Other studies also showed Th2 and a mixed Th1/Th2 response after nanoparticle immunization. Besides the importance of the challenge assay with infectious viruses on immunized mice, few studies used this approach to validate the potential of nanovaccines to protect animals from infection [61,62,63,64,65,66,70,71,72,75,76,77,78,80,81].

### 2.3. Limitations about the Use of Nanoparticles to Prevent Arboviruses Infections

Although there is a clear rational basis for the use of nanoparticle-based vaccines to prevent arbovirus infection, the lack of methodology standardization among studies is a huge weakness in this field. Some studies did not report important data about nanoparticle formulation and physical-chemical nanoparticle characterization, such as size, morphology, zeta potential, and encapsulation or adsorption rate. Some in vitro and in vivo analysis were also neglected. For example, the cytotoxicity and the interaction of nanoparticle-based vaccines with antigen processing cells and other immune cells should be among the main investigation subjects. It is imperative to understand if these nanoparticles could induce some crucial players’ activation in innate and adaptive immune responses, such as dendritic cells and T cells, respectively. Additionally, many studies failed to perform a challenge assay in nanoparticle immunized mice.

### 2.4. Roadmap Proposal

**Figure 4:**
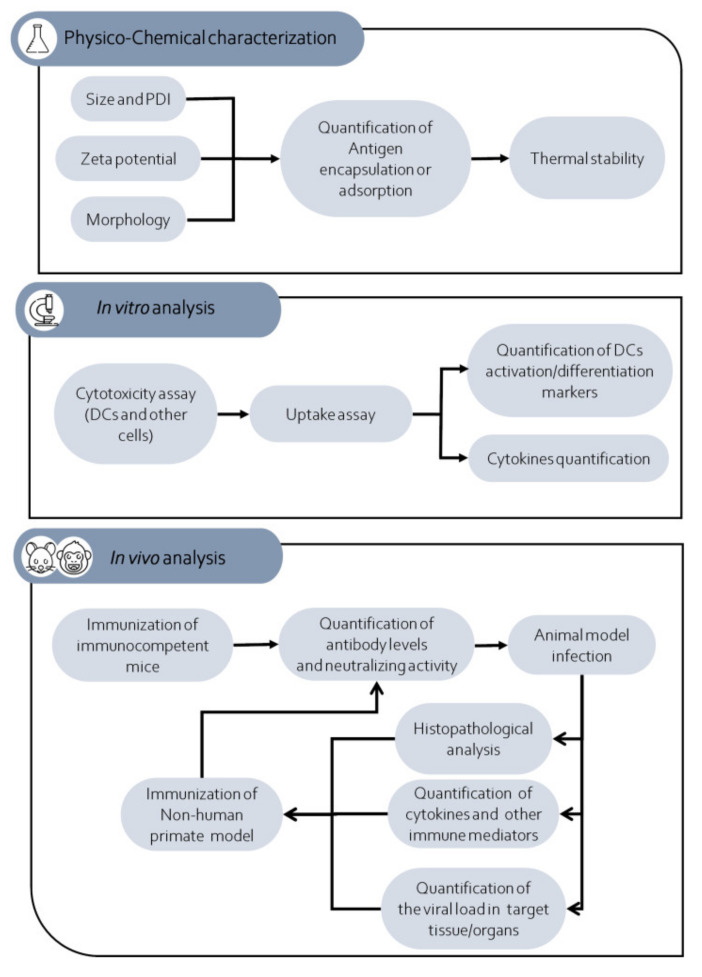
Schematic representation of the experimental steps comprised in the proposed roadmap. Legend: DCs: Dendritic cells; PDI: polydispersity index.

Therefore, we propose a roadmap that can help researchers to develop and evaluate the potential of nanoparticles to induce a protective immune response against an arbovirus (Figure 4). The first set of experiments should be done to characterize the new nanovaccine candidate. The research should measure the size, surface charge (zeta potential), polydispersity index, and morphology of the proposed vaccine candidate. Antigen encapsulation or antigen adsorption rate is also important. Another important aspect to be evaluated is the thermal stability of these nanoparticles at different temperatures (low, medium, and high). Performing experiments using low (−20 to 4 °C) and moderate temperatures (10–25 °C) that mimic vaccine transport and storage could be very useful to determine the stability of this nanovaccine at field conditions. Additionally, using high temperatures (26 to 40 °C) could be important to gain insights about using these nanovaccines on countries and regions with high annual temperatures and without a proper cold chain to transport, store, and handle vaccines from the manufacturer to the administration of the vaccine. In vitro assays that aim to obtain information about the degradation rate, antigen release, and the physical characteristics of these nanoparticles at different temperatures are crucial to determining the best way to produce, transport, and store these vaccine candidates [109,110,111].

The second set of experiments is essential to characterize the nanoparticles’ in vitro effect on antigen-presenting cells, such as dendritic cells and macrophages. Besides, cytotoxicity assay of nanoparticles is crucial to identifying these nanoparticles’ impact on cells and determining if they are non-toxic and well-tolerated by them [82,112]. Additionally, other cells (primary cells or cell lines) could be tested. Concerning the antigen-presenting cells, it is important to verify whether the nanoparticle treatment could increase the phagocytic, antigen processing, and antigen-presenting ability of these cells. Biological assays that aim to quantify cytokines (e.g., type I interferon, IL-6, and TNF-alpha) and activation markers (e.g., CD86/80, MHC-I, MHC-II, and CD40) are also important [69]. However, other parameters such as the effect of nanoparticles on organelles and cellular morphology could be also evaluated depending on the researcher´s objectives. 

Once data obtained by previous analysis demonstrated an optimal antigen association/encapsulation rate, a thermal stability, the absence of cytotoxicity on cells and an increase of phagocytic, antigen processing, and antigen-presenting ability of antigen presenting cells, the research could initiate the in vivo preclinical assays. When it does not, research should come back to nanoparticle design and try to modify its structure to get an improved immunogenic and antigenic effect of nanoparticles on antigen-presenting cells. Mice should be the first animal model used to evaluate the in vivo immunogenicity of nanoparticles. Researchers must be careful in the choice of mouse strain. Knockout-out mice (e.g., type I IFN receptor) should be avoided in the immunization assays, and immunocompetent mice should be immunized in single or multiple dose experiments. After the immunization protocol, sera from these mice should be obtained to measure IgG and the neutralizing activity of these antibodies against the target arbovirus.

The most critical step in this proposed roadmap is the infection of immunized mice with the arbovirus. As mentioned above, many arboviruses cannot replicate in immunocompetent mice, and therefore, this contributes to the difficulty of studying arbovirus replication and pathogenesis in these mice [103,108]. Therefore, measuring the role of a nanoparticle-induced immune protective response in immunocompetent mice after arbovirus infection is an obstacle for many researchers and laboratories. A possible way to overcome this is to use an immune depletion strategy before arbovirus infection. Some studies have been shown that injection of antibodies against type I interferon receptors one or more days before arbovirus infection could facilitate virus infection, dissemination, and pathogenesis [100,107]. Therefore, this strategy could generate data about the role of pre-existing immunity (antibodies, TCD4, TCD8, and B memory cells) in the context of an arbovirus infection. 

In challenge assays, it is important to evaluate the clinical signs in the infected mice (weight and mice parameters behaviors) [107,113]. Quantification of the viral load in the target tissues and organs is imperative [101,103,114,115]. Histopathological analysis of these tissues/organs should complement the analysis and provide insights about reducing the cytopathic effect on the immunized mice compared to non-immunized mice. Serum and tissue cytokines could also be quantified. These important immune mediators’ expression is important to define the type of T cell response (Th1, Th2, or Th17) induced in the infected immunized mice [65]. Additionally, analysis of TCD4 and TCD8 activation markers could be measured by flow cytometry in these animals to generate information about the T cell response on immunized animals after infection. Once all data indicate the production of neutralizing antibodies and/or T cell response that are able to reduce clinical signs, viral load and also damage on target tissues/organs efforts should be undertaken to test this nanoparticle on NHP models. This is the most challenging step in this proposed roadmap because of the high cost of these preclinical models and the low number of institutions able to properly conduct this test [103]. Since mouse and NPH models are different, researchers should provide adequate nanoparticle dose to NHP and also verify what immunological and biological parameters are possible to evaluate in this model. In general, quantification of neutralizing antibodies is a good marker of successful immunization on the NHP model. When possible, the immunized monkeys should be challenged with an arbovirus, and the same parameters described for mice should be measured on these monkeys. However, researchers should consider that some arbovirus NHP models shown a low viremia and an absence of clinical signs. 

## 3. Conclusions

In conclusion, considerable research has been undertaken to develop and evaluate nanovaccines against DENV, ZIKV, JEV, WNV, and CHIKV. However, we did not find any study that aimed to develop nanovaccines against other important arboviruses (RVFV, OROV, and MAYV). Nevertheless, the results presented here show us that, even with great advances in this field, we still need to invest more significant efforts to address the nanoparticles’ potential to act as vaccines against these arboviruses. Thus, we proposed an experimental roadmap to help researchers better characterize and evaluate nanovaccines against medically important arboviruses.

## Data Availability

No new data were created or analyzed in this study. Data sharing is not applicable to this article.

## Supplementary Materials

The following are available online at https://www.mdpi.com/2076-0817/10/1/36/s1, Supplementary Methodology; Table S1; Figure S1: The number of studies that used nanotechnology to develop new vaccines against medically important arboviruses.
