# Supplementary material for: Nanoparticles as Vaccines to Prevent Arbovirus Infection: A Long Road Ahead
Source: Pathogens. 2021 Jan 5;10(1):36. doi: 10.3390/pathogens10010036 (PMC7824877; doi:10.3390/pathogens10010036)
Supplement: Supplementary file 1 [file pathogens-10-00036-s001.zip › Supplementary Materials_01.doc]

Supplementary Materials

1.Methodology

*1.1 Literature search*

A comprehensive bibliographic survey completed on 11/21/2019 at 6:00 PM was conducted in Scopus, PubMed, and Medline (https://www.ncbi.nlm.nih.gov/pubmed). The search strategy for original articles was composed of two components: a) nanoparticles; and b) some medically important arbovirus (Dengue virus, Zika virus, Japanese encephalitis virus, West Nile virus, Chikungunya virus, Rift Valley fever virus, Oropouche virus, and Mayaro virus).

*1.2. Data extraction and management*

Researchers independently performed the initial screening of the records retrieved from each database. Publication data (name of authors, journal, year, title, and abstract) were analyzed and duplicate registers removed. This study includes only original in vivo studies that investigated the use of nanotechnology to develop new vaccines against medically important arboviruses (DENV, ZIKV, JEV, WNV, CHIKV, RVFV, Oropouche virus (ORPV), and MAYV. The exclusion criteria used were: (1) secondary studies (e.g. literature review, letters to the editor, editorials, and comments); (2) gray literature (studies not subject to peer review or not published in formally indexed scientific journals); (3) incomplete articles; (4) in vitro studies; and (5) articles that did not use nanotechnology to develop new vaccines against arboviruses.

Data concerning nanoparticle characterization (material, size, potential zeta, and morphology), type of viral antigen (structural or non-structural antigens), type of vaccine (attenuated virus, inactivated virus, subunit, peptide, recombinant viral or bacterial vector, RNA, DNA, virus-like particle, conjugated vaccine), in vivo animal model used (mouse, monkey, human, other), and immune response induced after immunization protocol (antibody class, the effector function of antibodies, T cell activation, cytokines) were recorded on a spreadsheet for further analysis.

*1.3. Network construction*

The networks presented in this work were built using Gephi version 0.9.2 [22]. The components of each graph were listed in a comma-separated values (.csv) spreadsheet for each graph, and this file was imported into the software. Another .csv spreadsheet with the connections between the components was also imported to generate the network graph. In all networks, the node diameter was directly proportional to the edge degree. The layout was generated using algorithms based on the force of attraction and repulsion of the nodes (Force Atlas 2). The nodes were submitted to local rearrangement for better visualization of the connections between them. Appendix B

All appendix sections must be cited in the main text. In the appendixes, Figures, Tables, etc. should be labeled starting with ‘A’, e.g., Figure A1, Figure A2, etc.

**Appendix B**

**Supplementary Figure 1. The number of studies that used nanotechnology to develop new vaccines against medically important arboviruses**. CHIKV: Chikungunya virus; DENV: Dengue virus; JEV: Japanese encephalitis virus; MAYV: Mayaro virus; ORPV: Oropouche virus; RVFV: Rift Valley fever virus; WNV: West Nile virus; ZIKV: Zika virus.
